# Methodological landscape in the field of integration site identification of retroviruses and retroviral vectors

**DOI:** 10.3389/fbioe.2025.1708724

**Published:** 2025-11-11

**Authors:** Konstantin Kochergin-Nikitsky, Alexander Lavrov, Svetlana Smirnikhina

**Affiliations:** Laboratory of Genome Editing, Research Centre for Medical Genetics, Moscow, Russia

**Keywords:** retroviral vectors, provirus mapping, integration site, gene therapy, insertional mutagenesis, clonal expansion

## Abstract

Detailed mapping of viral vector integration sites, including retroviral and particularly lentiviral vectors, is critical for assessing their safety in preclinical and clinical studies. Although integration into the host genome follows certain virus-specific patterns, it remains a stochastic event and can cause insertional mutagenesis with diverse consequences, such as oncogene activation. In this review, we trace the evolution of ISA methods applied to retroviruses and derived vectors, from early labor-intensive approaches with limited coverage—such as combined strategies involving restriction analysis, Southern blotting, and subcloning—to modern high-throughput strategies. We discuss key methodologies that shaped the field, including inverse PCR (iPCR), ligation-mediated PCR (LM-PCR), and linear amplification-mediated PCR (LAM-PCR), highlighting their contributions to more comprehensive and unbiased mapping, along with limitations associated with systematic errors stemming from dependence on restriction endonuclease digestion and amplification biases. We also examine recent approaches designed to overcome these limitations, independent of PCR and restriction analysis, which enable a more accurate and undistorted representation of retroviral vector integration profiles. Despite the emergence of new techniques, classical methods—particularly LAM-PCR and its modifications, such as nrLAM-PCR—remain widely used and continue to serve as the standard in many commercial platforms.

## 1 Introduction

Personalized medicine is a key field in the development of modern medicine. In turn, controlled modification of the genetic context provides the basis for many of its approaches. While delivering transgenes into cell lines using plasmids and methods such as lipofection or electroporation poses no major challenge, recombinant viral vectors (VVs) are valuable tools for delivering genetic constructs into cells of multicellular organisms due to their evolutionarily developed adaptations facilitating infection of specific cells and tissues, including viral tropism and host cell entry mechanisms. Among different types of recombinant VVs, retroviral VVs have the intrinsic ability to maintain prolonged expression of exogenous genetic agents, integrating the viral genome into the genome of the host cell (proviruses). At the same time, the aforementioned capability is obviously potentially dangerous. For retroviruses, including lentiviruses, integration, while showing some preference for certain types of genomic regions, is not strictly targeted. It is considered that lentiviruses tend to prefer intragenic loci, whereas other commonly used retroviral vectors show a preference for 5′ transcriptional regulatory regions (Vijaya et al., 1986; Scherdin et al., 1990; Schröder et al., 2002; Hanai et al., 2004; Kok et al., 2024; Daniel and Smith, 2008; Yoder et al., 2021; Shao et al., 2022). Although gene knockout caused by lentivector insertion is usually not the goal of the manipulation, it can be less detrimental than the occasional random activation of oncogenes resulting from retroviral vector integration in regulatory regions (Corcoran et al., 1984; Hacein-Bey-Abina et al., 2003a; Ye et al., 2023). This observed tendency makes integrating lentiviral vectors (LVVs) more acceptable for drug development without the need for exhaustive genome sequencing of every transduced cell, while not alleviating the necessity of stringent control measures. Notably, early SCID-X1 gene therapy trials using γ-retroviral vectors aimed to restore functional T cells by *ex vivo* transduction of hematopoietic stem cells, followed by autologous transplantation, which led to LMO2-associated clonal T-cell proliferation in several patients. This revealed the severe oncogenic risks of untargeted retroviral integrations. Detailed analysis of these cases showed that the insertional activation of proto-oncogenes could drive clonal dominance even when only a small fraction of transduced cells were affected. This critical observation directly motivated the development of comprehensive, unbiased integration-site analysis (ISA) methods, enabling systematic monitoring of clonal expansion and identification of potentially oncogenic integration events, which is considered essential for all types of integrating vectors, particularly in preclinical studies, and in the case of retrovirus-based vectors, it often requires genome-wide analysis (Hacein-Bey-Abina et al., 2003a; Hacein-Bey-Abina et al., 2008). In most cases, the seemingly straightforward approach to IS identification—whole-genome sequencing (WGS) of transduced cells—proves impractical as achieving the required sensitivity when DNA from pooled cell samples is being sequenced demands extremely high coverage (by WGS standards), making the analysis prohibitively expensive and laborious. Moreover, in most use-case scenarios involving integrating vectors—whether during *in vivo* administration or *ex vivo* cell modification followed by autologous transplantation—sequencing the entire population of transduced cells would imply sacrificing the treated organism or the transplant itself. Therefore, at the preclinical stage, methods designed to ensure a maximally representative description of integration events are usually applied, focusing on the general profiling of integrations and the identification of their hot spots specific to the viral vector under investigation.

## 2 Early approaches to identification of insertions

Before the advent of automated sequencing and, later, WGS (late 1970s–1980s), the analysis of proviral integration relied mainly on restriction endonuclease analysis (REA) combined with Southern blot (SB), which enabled the detection of specific sequences up to several tens of kb and the estimation of the proviral load (Chan and Martin, 1980). Using probes in provirus-flanking regions, integrations could be mapped within preselected loci (Vijaya et al., 1986; Scherdin et al., 1990; Selten et al., 1984; King et al., 1985; Lavu and Reddy, 1986; Robinson and Gagnon, 1986; Isfort et al., 1987; Lobel et al., 1989; Ben-David et al., 1990), leading to the description of structural features such as LTR redundancy (Hughes et al., 1978; Varmus et al., 1981) and the identification of oncogenic hotspots such as *INT1/WNT1* (Nusse and Varmus, 1982). Molecular cloning and local sequencing of host–virus junctions (Lobel et al., 1989; van Ooyen and Nusse, 1984; Frankel et al., 1985; Shih et al., 1984; Wolf and Rotter, 1984; Swift et al., 1987; van Lohuizen et al., 1989; Clurman and Hayward, 1989) increased the resolution, revealing both dispersed and recurrent insertions, including those activating c-*Myc* (Corcoran et al., 1984) or disrupting *B2M*, Mov-13, and *INT1* (van Ooyen and Nusse, 1984; Frankel et al., 1985; Harbers et al., 1984).

In the 1990s, the methodological toolkit expanded with S1-mapping, shuttle vector systems (Cepko et al., 1984), and hybridization approaches such as IS-phage libraries, which demonstrated RSV preference for AT-rich, DNase I-hypersensitive regions (Shih et al., 1988). Genetic strategies (backcrosses and co-segregation tracking) identified new common integration sites (*EVI-5* and *NOTCH1*) (Tsichlis et al., 1990; Buchberg et al., 1999; Liao et al., 1995; Girard et al., 1996; Tam et al., 1997; Blaydes et al., 2001), while expression analyses (Northern blot and transcript sequencing) confirmed the functional impact of insertions, e.g., in *NOTCH1* (Lee et al., 1999; Yanagawa et al., 2000), and the tendency of M-MuLV to integrate into actively transcribed regions (Mooslehner et al., 1990). Dideoxy sequencing of distorted DNA regions provided early evidence of integration bias toward curved DNA (Pryciak and Varmus, 1992; Müller and Varmus, 1994; Pruss et al., 1994), signaling the gradual methodological shift of the 1990s toward PCR-based and more systematic sequencing approaches (Pryciak and Varmus, 1992; Müller and Varmus, 1994; Pruss et al., 1994; Benkel et al., 1992; Gong et al., 1998).

By the early 2000s, classical methods were increasingly becoming insufficient and lacked sensitivity and throughput, particularly for polyclonal samples or large-scale safety assessment of vector integrations (Tsichlis et al., 1990; Buchberg et al., 1999; Liao et al., 1995).

## 3 PCR-based recovery of non-predicted IS with downstream sequencing

Non-targeted strategies for isolating proviruses together with their flanking host sequences, enabling unbiased mapping of integration sites and subsequent sequencing, began to emerge in the 1990s, reflecting a methodological shift driven by the need for more comprehensive and detailed analyses of retroviral integrations. Various PCR-based techniques were utilized, such as inverse PCR (iPCR), ligation-mediated PCR (LM-PCR), splinkerette-PCR, vectorette-PCR, LAM-PCR, and nrLAM-PCR

IPCR. This PCR variation was developed as a “genome-walking” approach, allowing investigation of the genome “terra incognita” starting from regions with known sequences. It was presumably invented independently and simultaneously by several groups. Triglia et al. (1988) and Ochman et al. (1988) proposed essentially the same method, enabling amplification of sequences “that lie outside the boundaries of known sequences” (c), named by the latter as “inverse” PCR. One year later, Silver and Keerikatte (1989) proposed a similar approach, allowing the isolation of provirus-adjacent regions and having the potential to significantly accelerate the analysis of proviral integration sites.

**FIGURE 1:**
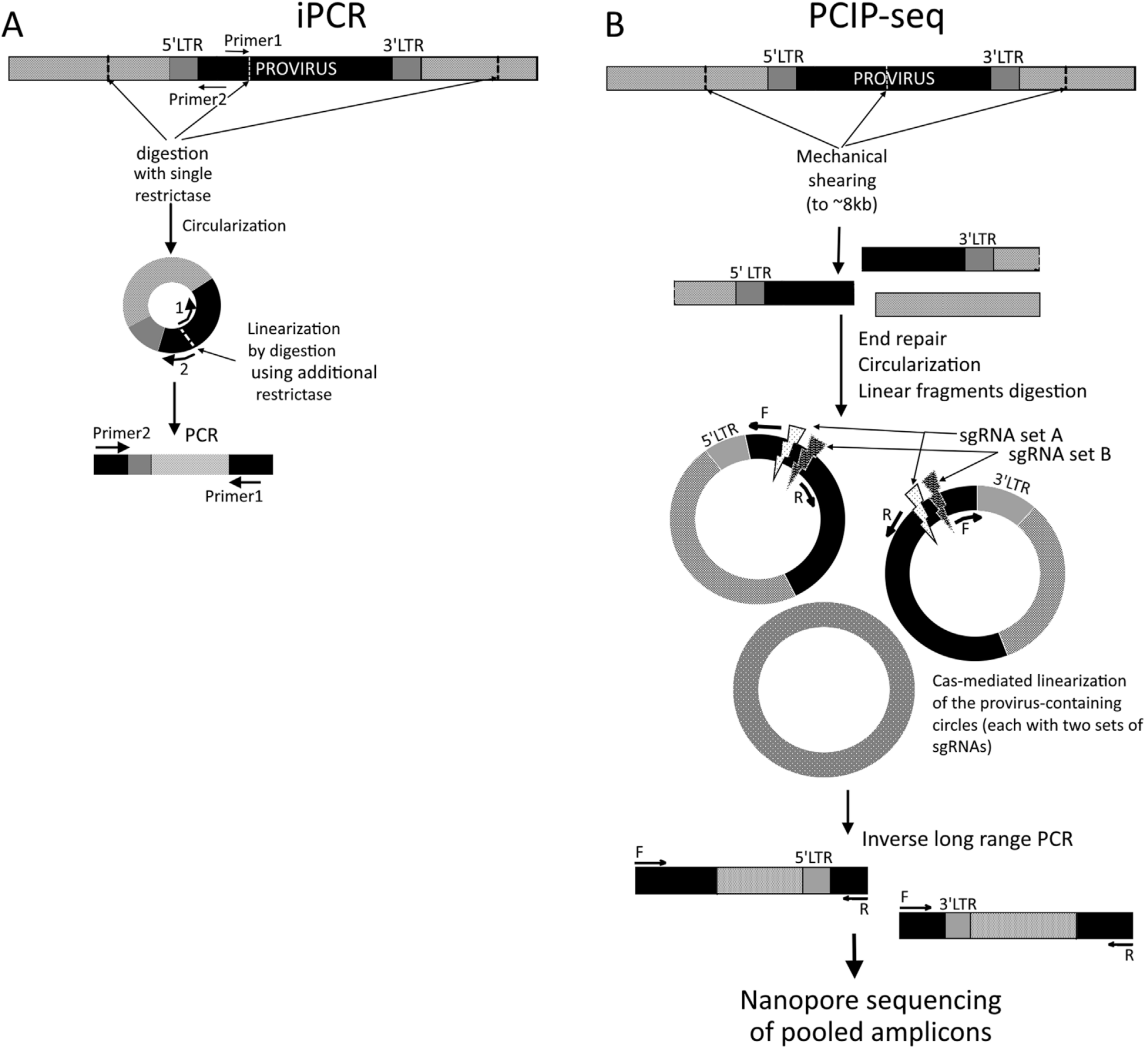
Schematic representation of the iPCR **(A)** and PCIP-seq **(B)** methods. Black boxes represent known sequences (proviral), while light-gray boxes represent genomic DNA. **(A)** 5′ LTR-containing circles are shown. Optional linearization is performed using another restriction enzyme with a cut site between 5′ ends of primers.

The method is based on a simple yet practical idea. Standard PCR requires known flanking regions for primers to anneal. If there is a known sequence (provirus in the context of ISA) surrounded by uncharted DNA, this known island can be turned “inside out” so that the unknown flanking sequences (or sequence, if the second cut site is within the provirus) become flanked by parts of the known region, enabling simple two-primer exponential PCR. To do this, the unknown sequences are cut at some distance and circularized using DNA-ligase. Then, primers placed in the known region with their 5′ ends facing each other and 3′ ends directed into the terra incognita allow conventional PCR on the circular template (Figure 1A). To improve the efficiency of such PCR, it is also recommended to cut this circular DNA somewhere between the 5′ ends of primers. In the absence of sequencing methods distinguishing individual reads, the natural approach was to subclone single amplicons and perform Sanger sequencing on each clone, thereby obtaining two deciphered flanks for each known cutout isle. A notable complication (and prolongation) in using this method arises from the fact that achieving efficient monomeric ligation rather than concatemers requires careful empirical adjustment of fragment concentrations (despite the theoretical rationale described) and is complicated by the widely varying fragment sizes (Green and Sambrook, 2019).

Being less time- and labor-intensive, more sensitive, and allowing far more comprehensive ISA than earlier locus-limited methods, iPCR was widely applied in the late 1990s and 2000s to study clonal properties of host cells infected with integrating viruses such as HTLV-1, STLV-1, HIV-1, MMLV, and even HBV or mobile elements, enabling the detection of clones at <1% frequency (Hanai et al., 2004; Takemoto et al., 1994; Cavrois et al., 1995; Ohshima et al., 1997; Kikuchi et al., 1997; Gabet et al., 2003; d’Offay et al., 2013). Precise mapping of insertion sites (ISs) revealed non-random integration patterns influenced by chromatin, gene proximity, or sequence motifs, with biases such as avoidance of centromeric repeats (HIV-1), targeting of proto-oncogenes (MMLV and HTLV-1), or conserved insertion hot spots (IncJ in *E. coli*), supporting adaptive or context-dependent integration mechanisms (Hanai et al., 2004; Cartea et al., 1998; Li et al., 1999; Mack et al., 2003; McGrath and Pembroke, 2004; Zhu et al., 2012).

### 3.1 LM-PCR/LAM-PCR/nrLAM-PCR

#### 3.1.1 LM-PCR

A real breakthrough came with the LM-PCR method, introduced in the last 1980s to early 1990s (Pfeifer et al., 1989; Garrity and Wold, 2025; Mueller and Wold, 2025), which became widely adopted by the early 2000s as a core technique for mapping proviruses. In the context of mapping proviruses, this approach involves “blind” ligation of short adapters to restriction-digested host genomic DNA, including fragments containing viral insertions. PCR primers are typically designed so that one anneals to the viral sequences and the other anneals to the ligated adapters, enabling simultaneous amplification of many distinct DNA fragments in a single reaction with a common primer pair or two pairs in the nested PCR variant. This allows potential amplification of a large number of fragments. Without additional cloning, this method is suitable for creating libraries for high-throughput sequencing (next-generation sequencing, NGS) as it allows the observation of individual reads. However, NGS was not yet available, and researchers typically relied on automated Sanger capillary sequencers, which limited the method’s capacity and required cloning of amplicons to separate products amplified from different loci.

The LM-PCR-based ISA approach enabled, in one of the early foundational studies by Schröder et al. (2002), the detailed characterization of HIV-1 and recombinant viral vector integration specificity, resulting in 524 mapped integration sites in SupT1 cells and 111 *in vitro* control sites, revealing a preferential integration into active gene bodies. Subsequently, similar approaches were applied in clinical gene-marking studies (Wehnert et al., 2004), which revealed the leukemogenic potential of SIN vectors with strong enhancers integrating near critical targets such as EVI1 or PRDM16 (Correa de Freitas et al., 2014) and others. Since the mid-2000s, some NGS techniques, starting with pyrosequencing, began to be incorporated into LM-PCR-based ISA pipelines, allowing the avoidance of amplicon subcloning and significantly increasing the throughput of ISA (Hacein-Bey-Abina et al., 2008; Biffi et al., 2011; Dawes et al., 2020). For example, recently, Welles et al. (2022) showed ongoing clonal expansion of infected cells despite antiretroviral therapy (ART) (Rozera et al., 2022).

With the implementation of NGS techniques, restriction endonuclease digestion (RED) in some ISA protocols has been replaced by random DNA shearing via sonication. This alleviated RED-dependent limitations such as limited restriction site availability, causing incomplete or biased coverage, site-dependent blind spots where integrations distant from restriction sites remain undetected, and the overall loss of proviral insertions. Simultaneously, it simplified and expedited the workflow, rendering the methods more versatile, sensitive, and better suited for high-throughput applications (Hacein-Bey-Abina et al., 2008; Gillet et al., 2011; Berry et al., 2012; Serrao et al., 2016; Wells et al., 2020). A representative example is the approach used by Justice et al. (2015), who applied sonication-based fragmentation followed by adapter ligation and nested PCR with virus-specific and adapter-specific primers to selectively amplify virus–host junctions, preparing libraries for Illumina high-throughput sequencing, which enabled mapping of 32,050 unique ALV integration sites *in vivo* and the analysis of integration clusters and clonal expansion.

Non-perfect conversion of fragments processed in a single-primer reaction into the linker-ligated product should be kept in mind. There are mostly inevitable losses during the repair of DNA ends with incompatible overhangs, nicks, or missing phosphates (generated by sonication) and, in protocols such as Illumina library preparation, during A-tailing (Serrao et al., 2016). Nevertheless, an LM-PCR-based approach has gained widespread adoption. It has been applied, for example, to detect T-DNA insertions in the genome of *Arabidopsis thaliana* arising from agrobacterium-mediated transformation (O’Malley et al., 2007). Moreover, there are commercially available kits for IS identification, in which libraries are prepared using LM-PCR, such as the Retro-X Retrovirus Integration Site Analysis Kit, which is available online at https://www.takarabio.com/about (TaKaRa Bio, Japan).

#### 3.1.2 Important LM-PCR derivatives

One recent LM-PCR modification is cassette-ligation PCR. Here, the conventional adapter/linker is replaced with a cassette of two partially complementary strands: a long strand (∼27 nt) serving as a primer template and a short strand (∼14 nt) with a 3′-end mismatch, preventing nonspecific priming. The 3′-ends of genomic fragments are blocked with ddCTP before ligation to reduce the background. This design provides more uniform and accurate amplification of integration sites. Zhang et al. (2020) combined this method with nanopore sequencing, using RED-based digestion with NcoI and BspHI (cutting throughout the provirus but not within LTRs), significantly reducing amplification bias compared to that with inverse PCR and classical LM-PCR, and enabling representative capture of polyclonal sites after T-cell gene therapy. Coupling with nanopore sequencing allows high-throughput, sensitive detection of thousands of unique integration sites with low background (Zhang et al., 2020).

Vectorette-PCR and splinkerette-PCR. Vectorette- and splinkerette-PCR are LM-PCR variants with modified adapters, originally developed for genome walking. Vectorette-PCR was developed in the early 1990s (Arnold and Hodgson, 1991), and splinkerette-PCR was developed around 1994 (Qureshi et al., 1994). Both use complex adapters, which are designed so that primers cannot anneal directly to the adapter itself, ensuring that amplification occurs only from newly synthesized strands containing viral and flanking genomic sequences. In vectorette adapters, a central non-complementary region reduces the background, while splinkerette adapters form a polymerase-blocking hairpin on one strand at all stages except at denaturation, due to a self-complementary region. In splinkerette-PCR, only one adapter strand can cause nonspecific priming, whereas in vectorette-PCR, both strands may still allow erroneous priming.

Apparently, due to the greater specificity and structural stability of the adapter, the splinkerette-PCR variant has largely replaced the vectorette-PCR over time. In particular, in the field of viral integration-site detection, splinkerette-PCR became relatively widespread in the 2010s (Dambrot et al., 2014; Uren et al., 2009; Yin, 2011; Šenigl et al., 2012; Shao and Lok, 2014; Jong et al., 2014; Šenigl et al., 2017; Potter and Luo, 2010; Lenvi et al., 2002; Lund et al., 2002), while the number of studies using vectorette-PCR remains limited (Cartea et al., 1998; Allen et al., 1994; Sfanos et al., 2011).

#### 3.1.3 LAM-PCR

Schmidt et al. (2001) presented the first demonstrated modification of LM-PCR, which later evolved into the so-named LAM-PCR, specifically adapted for high-sensitivity mapping of retroviral integrations. They developed a method called EPTS/LM-PCR (magnetic extension primer tag selection/ligation-mediated PCR), which significantly expanded the capabilities for finding integration sites (Schmidt et al., 2001). The key modifications involved the inclusion of an IS surrounding-independent linear amplification stage and an enrichment stage—EPTS. A biotinylated virus-specific primer complementary to the LTR region was used for single-primer extension. The products, containing a biotin tag, could then be selectively captured by streptavidin-coated magnetic beads after the removal of unused primers, with the washing away of non-target genomic DNA and restriction digestion of the extension products. The approach provided high sensitivity, allowing the detection of proviral sequences at low copy numbers: down to one copy per 100–1,000 cells in complex biological samples. In this case, nucleotide sequences were obtained for individual identified amplicons via cycle sequencing. With minimal modification, the approach originally proposed by Schmidt et al. (2001) was adopted by other groups (Laufs et al., 2003; Varas et al., 2009).

**FIGURE 2:**
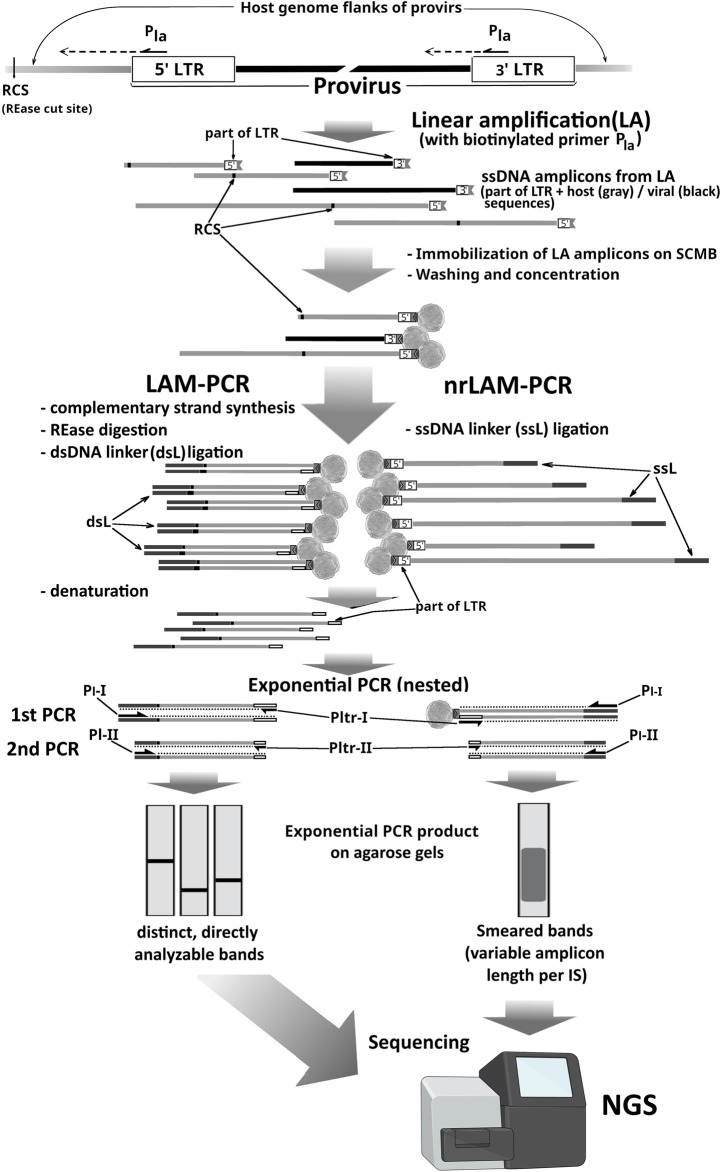
Comparison of LAM-PCR and nrLAM-PCR. LA, linear amplification reaction; Pla, biotinylated primer used in LA; RCS, digestion sites for the chosen REase; Pltr-I/II, LTR-complementary primers for exponential PCR (expPCR); Pl-I/II, linker-complementary expPCR primers; ssL/dsL, linker cassettes (dark gray lines); gray lines, host genome sequences; black lines, internal viral sequences; black-bordered white boxes, 3′LTR or 5′ LTR part of amplicons.

The method and name LAM-PCR were first introduced by Schmidt M et al in 2003 and later in 2007 and 2009 (Woods et al., 2003; Schmidt et al., 2007; Schmidt et al., 2009) by building on earlier strategies to develop a highly sensitive technique for identifying proviral insertions in the host genome. Like earlier EPTS/LM-PCR, LAM-PCR involves restriction digestion, single-primer extension using a biotinylated primer, and ligation of a known adapter to unknown genomic DNA fragments flanking the provirus–host junctions. However, the sequence of steps in LAM-PCR differs. After multicycle LA with a biotinylated primer complementary to the viral sequence and solid-phase enrichment of target sequences on streptavidin-coated beads, the immobilized single-stranded DNA is enzymatically converted to double-stranded DNA. This double-stranded DNA is then restriction-digested, ligated to a double-stranded linker of known sequence, and subjected to nested PCR using primers that are specific to the viral region and the linker (Figure 2). The subsequent steps are similar to those of the standard LM-PCR approaches: either cloning into plasmids for Sanger sequencing or direct sequencing using NGS-based methods.

Since the early 2000s, the LAM-PCR method has been implemented in numerous studies without significant modifications. It has been widely applied to identify retroviral integration sites in various experimental and clinical contexts, including the transplantation of transduced hematopoietic stem cells (Gonzalez-Murillo et al., 2008; Hayakawa et al., 2009; Montini et al., 2009; Mitsuhashi et al., 2010; Rong et al., 2017). As a relevant method, LAM-PCR was subsequently adapted for use with high-throughput sequencing, starting with pyrosequencing and extending to modern platforms such as Illumina (Cattoglio et al., 2010; Cornils et al., 2013; Zhang et al., 2013). An improved pipeline for bioinformatical analysis of the NGS results, acquired using LAM-PCR, was proposed by Rosewick et al. (2020). This pipeline provided faster, more accurate, and reproducible identification of integration sites by replacing BLAST with Bowtie2 for read mapping, automating event filtering and clustering, and compensating for underrepresentation of GC-rich regions typically caused by PCR and NGS-related GC bias (Rosewick et al., 2020).

Despite its high sensitivity and specificity, LAM-PCR remains methodologically complex, requiring primer biotinylation, capture steps, and adapter ligation. Its performance depends on the genomic distribution of restriction sites, which limits its universality. These limitations prompted the development of new approaches that overcome the drawbacks of LAM-PCR and offer greater flexibility in application. As early as 2007, criticism of the LAM-PCR method was expressed by Harkey et al. (2007), who noted that standard LAM-PCR fails to detect 30%–40% of clones even after thorough analysis and that the relative abundance of specific clones in a mixture can be distorted by up to 60-fold, which severely limits quantitative assessment and reliable clonal tracking. Additional limitations included the labor-intensive nature of the method, the need for large sequence databases that are difficult to generate using the existing approach, and the dependence on restriction-site availability, which can suppress the detection of certain integration sites. As an alternative, the authors proposed a modification involving the use of multiple restriction enzymes to account for random clustering and systematic bias, along with an extra digestion step targeting the recognition sites within the provirus, followed by the removal of cleaved fragments to eliminate non-informative internal amplicons. According to the authors, these improvements increased the global detection capacity to over 90%, reduced systematic errors, and improved the accuracy of clonal quantification.

#### 3.1.4 nrLAM-PCR

With the expanding use of recombinant retroviral vectors in gene therapy and, particularly, following reports of insertional activation of proto-oncogenes leading to clonal outgrowth and leukemogenesis in clinical trials (Hacein-Bey-Abina et al., 2003a; Hacein-Bey-Abina et al., 2003b; Ott et al., 2006; Howe et al., 2008), the need for comprehensive, genome-wide mapping of IS has gained significant demand. The identification of integration loci with high sensitivity and minimal bias is essential for understanding vector–host interactions and ensuring the safety of integrating vector systems.

By 2009–2010, all three widely used ISA PCR-based approaches were inverse PCR (iPCR), ligation-mediated PCR (LM-PCR), and linear amplification-mediated PCR (LAM-PCR), which shared a fundamental limitation determined by their RED-dependency for the extraction of the provirus–genome junctions. The positioning of some integration sites relative to the recognition sites of the selected restriction enzymes could be suboptimal; the recognition sites themselves are unevenly distributed throughout the genome (especially in heterochromatic regions), and some integration sites might be located in regions less accessible to restriction enzymes. All of this introduced a method-specific bias in the extraction of integration sites, leading to the underrepresentation of many integration events (Dawes et al., 2020; Schmidt et al., 2007; Harkey et al., 2007). Later, with the growing implementation of NGS, several of these methods were adapted to use mechanical DNA fragmentation—typically via sonication—as a more random and restriction-independent strategy; the inherent limitations of restriction-based fragmentation remained a concern during this transition period.

Thus, to address some of these concerns, the inventors of the LAM-PCR method proposed an improved alternative in 2009–2010: nonrestrictive linear amplification-mediated PCR (nrLAM-PCR) (an advancement over classical LAM-PCR) (Gabriel et al., 2009; Paruzynski et al., 2010), thus enabling genome-wide identification of IS without the need for restriction digestion. By removing the dependency on restriction enzymes, nrLAM-PCR directly addresses the site-specific bias of earlier methods, allowing a more uniform and representative recovery of integration sites across the genome. This improvement enhances the reliability of clonal analysis in gene-modified cells.

The nrLAM-PCR protocol starts from LA on genomic DNA with a single biotinylated primer annealing to the vector’s LTR. Amplification of the 3′ junction requires the primer to be positioned within the U5 region of the 3′ LTR, as close as possible to the host genome. Conversely, for the 5′ junction, it should be placed in the U3 region, since the LTR sequence—more than 600 nucleotides in the HIV-1 case—reduces the effective length of informative reads during sequencing. Similarly to LAM-PCR, biotinylated ssDNA products are enriched using streptavidin-coated beads. The key distinction lies in the unnecessary RED shearing of host DNA. Specific ssDNA-linkers (5′-phosphorylated and bearing a 3′-terminal dideoxycytidine) are ligated to the ssDNA products of linear amplification that serve as priming sites for the universal primer in the subsequent exponential PCR amplification (usually in nested variants) (Figure 2). Modification of the linkers prevents undesired ligation and ensures unidirectional attachment. The variable length of products from the LA stage prevents the resolution of individual junctions by AGE after PCR, so the sequencing is essential for clonality analysis, and an additional stage introducing platform-specific adapters is required (Wang et al., 2016). Another drawback of nrLAM-PCR-based ISA approaches is the lower sensitivity than LAM-PCR at low amounts of input DNA, while still being comparable to that of the LM-PCR-based approaches (Gabriel et al., 2009; Paruzynski et al., 2010; Wang et al., 2016; Paruzynski et al., 2012; Gabriel et al., 2014).

This method, proposed as a comprehensive methodological approach for highly sensitive and accurate analysis of the clonal repertoire of gene-corrected cells, including hematopoietic stem/progenitor cells (HSPCs) (Paruzynski et al., 2012; Giordano et al., 2015), has been in use since the 2010s. It was implemented for the ISA of integration events in transgenesis techniques using integrating vectors, such as piggyBac-based vectors (Marh et al., 2012), in studies of natural integrating viruses, including HIV-1 in the context of ART (Kok et al., 2024; Hauber et al., 2013; Einkauf et al., 2025), along with the safety assessment of retroviral (lenti- and γ-retroviral) vectors in autologous (Carbonaro et al., 2014; Zhang et al., 2022) or heterologous HSPC transplantation (Shi et al., 2014) in differentiating cell lines (Karumbayaram et al., 2012; Chin et al., 2016; Saito et al., 2020).

#### 3.1.5 Approaches aiming to improve uniformity in PCR-based IS recovery

In addition to the above-mentioned nrLAM-PCR, the bias introduced by the use of RED-dependent DNA shearing in standard ISA methods such as iPCR, LM-PCR, and LAM-PCR, related to the uneven distribution of recognition sites across the host genome and variable cleavage efficiency and, thus, the incomplete recovery of integration events, has been addressed in different approaches. For example, s-EPTS/LM-PCR (van Haasteren et al., 2021) and MGS-PCR were proposed as mechanical host DNA shearing-adapted variations of EPTS/PCR and LAM-PCR methods, respectively. Both methods implied NGS sequencing. In MGS-PCR (Beard et al., 2014), the key differences are the replacement of the standard ligatable adapter with a modified splinkerette bearing a 3′-T overhang complementary to the A overhang of the APE-PCR product, which enhances specificity and reduces nonspecific amplification, and the use of nanosized magnetic beads instead of standard magnetic beads (Xu et al., 2013).

MIP-seq. In 2019, in a study focused on intact HIV-1 proviruses accumulating at distinct chromosomal positions during prolonged antiretroviral therapy, Einkauf et al. (2025) proposed a comprehensive approach called MIP-seq, enabling the simultaneous identification of integrated proviral sequences and precise IS localization from genetic material derived from as few as a single cell. The main innovation was the addition to widely used pipelines based on LM-PCR and nrLAM-PCR of whole-genome nonspecific amplification of all available DNA in samples diluted to approximately one IS per reaction (i.e., down to single cells in some cases) via multiple displacement amplification (MDA) with phi29 DNA polymerase, characterized by high processivity, 3′→5′ exonuclease proofreading activity, and strand displacement capability. MDA is an isothermal amplification method wherein phi29 polymerase synthesizes multiple new strands while simultaneously displacing previously synthesized fragments (strand displacement) from random hexamer primers, resulting in branched and exponential DNA amplification without denaturation cycles. MDA-amplified genetic material was then split into two workflows (Vijaya et al., 1986): amplification of near full-length (∼8–9 kb) HIV-1 proviral sequences using specific primers and (Scherdin et al., 1990) analysis of integration sites using LM-PCR, nrLAM-PCR, or ISLA. Since both reactions were performed from the same MDA product derived from a single viral copy, it was possible to reliably link each sequenced proviral genome to its chromosomal integration site—a task previously achievable only with more laborious and less scalable methods. The method also proved useful for detecting and confirming clonal origins of individual integrated proviruses.

Another approach relying on MDA to improve sensitivity is the individual proviral sequencing assay (IPSA), which was proposed in 2022 by Joseph et al. (2022) as a method aimed at simultaneously determining the near-full-length structure of individual HIV-1 proviruses and their precise chromosomal integration sites in patients receiving antiretroviral therapy. Its workflow starts from MDA of genomic DNA using human genome-biased random decamers at 40 °C with trehalose to suppress nonspecific amplification. MDA products are purified with magnetic SPRI beads and split into two branches. In the first, nested PCR amplifies nearly full-length proviruses from the gag leader to the 3′ LTR. In the second, integration sites are identified using an LM-PCR-like NGS-adapted strategy: MDA products are digested with four restriction enzymes, end-repaired, A-tailed, ligated to adapters containing nullomers, and subjected to nested PCR using primers specific to both the adapter and the 5′ LTR, selectively amplifying host–provirus junctions. Amplicons from both branches are sequenced on the Illumina MiSeq platform. A key step is limiting the dilution of genomic DNA to a concentration at which fewer than 30% of the reactions are PCR-positive, ensuring that most wells contain no more than one provirus and allowing unambiguous linking of its structure to a specific integration site.

The method demonstrated high specificity, with an average of 41% of wells yielding informative results, thus linking individual provirus structures to their genomic integration sites. Limitations include low MDA efficiency due to endpoint dilution (a large fraction of reactions remains empty), complications related to deletions in primer-binding sites, a limited 49 bp overlap between NFL and IS amplicons, and absence of the final 69 bp of the 3′ LTR. The workflow is labor-intensive, requires manual screening, and is poorly scalable. When multiple proviruses occur in a single reaction (10%–15% of cases), data may become ambiguous, causing chimeric assemblies and misinterpretations.

Among other methods aimed at improving ISA uniformity, FLEA-PCR (Pule et al., 2008) strongly resembles nrLAM-PCR, with the main difference lying in the stage of the so-called “anchor primer” attachment to the products of linear amplification, which, in this approach, could include either a sequence external to the provirus (when the LA primer anneals within the 5′ LTR) or a section of the provirus sequence (within the 3′ LTR). This anchor primer includes a predefined 5′ part (used as a target for universal primers in the downstream PCR) and a random 3′ part, which is able to anneal to the unknown 3′ ends of LA stage products to prime dsDNA synthesis by either DNA polymerase I Klenow fragment or T7 DNA polymerase.

Approximately 23% of the sequenced clones contain only internal retroviral sequences and have to be excluded from analysis. In NGS workflows, bioinformatics pipelines can automatically filter such reads. The use of anchored primers with randomized 3′ tails may generate multiple amplicons from the same integration site, complicating data interpretation and requiring careful PCR optimization. FLEA-PCR was not widely adopted but has been used in a few studies relevant to this review, typically with NGS and protocol modifications (Henssen et al., 2015; Xu et al., 2023).

Recently developed RAISING, introduced in 2022 by a Japanese research team (Wada et al., 2022), is also a conceptual advancement of the nrLAM-PCR method. Unlike nrLAM-PCR, RAISING eliminates adapter ligation and the use of biotinylated primers, significantly reducing the assay time to approximately 3.5 h and lowering costs. A key feature of the method is the use of polynucleotide tailing (polyAG-tailing) combined with thermomodulation, enabling selective hybridization of a complementary oligo-dT adapter without the need for high-temperature denaturation and with controlled temperature ramping during annealing. This reduces the amplification of non-target genomic DNA regions, thereby increasing specificity and sensitivity. RAISING can detect integrations at a proviral load as low as approximately 0.03%, which is five times more sensitive than its predecessor RAIS, which itself was reported to be approximately 100 times more sensitive than nrLAM-PCR. The method is suitable for analyzing both monoclonal and polyclonal integrations and is compatible with Sanger sequencing and high-throughput sequencing (NGS). For Sanger data analysis, a specialized R package, CLOVA, with a web interface, is used to assess the clonality of integrated fragments.

Limitations of the methods include sensitivity to the quality of input DNA, requiring an optimal number of ssDNA synthesis cycles (25 cycles), and the use of the highly specific Q5 polymerase to ensure uniform and specific double-strand synthesis. Additionally, the method requires a column-based ssDNA purification step, which may affect the overall efficiency. Occasionally, nonspecific products may appear, necessitating careful PCR optimization.

In recent years, the iPCR methodology has advanced through a novel comprehensive approach called pooled CRISPR inverse PCR sequencing (PCIP-seq). Artesi et al. (2021) proposed it for the simultaneous determination of integration sites, proviral sequences, and clonal composition of infected cells in a single protocol using Oxford Nanopore long reads. This approach helps overcome the limitations of traditional iPCR-based methods with short-read sequencing as PCIP-seq enables the simultaneous identification of integration sites and near-complete provirus sequencing, along with RED-dependent bias. Genomic DNA from infected cells is mechanically sheared to ∼8 kb, typically producing two fragments per provirus (on average): one containing the 5′ end and upstream host DNA and the other containing the 3′ end and downstream host DNA. Fragments are then circularized using T4 ligase, and linear DNA is removed by a plasmid-safe ATP-dependent procedure. Selective linear cleavage of circular molecules is performed with Cas9 guided by pools of sgRNAs targeting multiple sites near the 5′ and 3′ LTRs. Two sets of sgRNAs and corresponding primers are used, designed so that each provirus fragment resulting from shearing contains a binding site for only one sgRNA-binding locus and a primer pair from each duplicate set. Primers are oriented outward from the cleavage site toward unknown sequences, enabling the amplification of regions in iPCR, including flanking host DNA and part of the provirus (Figure 1B). The resulting amplicons are purified, indexed, pooled, and sequenced on the Oxford Nanopore MinION. This design provides overlapping amplification of provirus regions and near-complete coverage.

The method demonstrated high sensitivity for detecting integration sites due to long reads, enabling precise mapping in complex repetitive regions and simultaneous retrieval of proviral sequence, clonality, and epigenetic information, but it is mostly limited by the need for large input DNA (3 µg), reducing its applicability for samples with low proviral copy numbers. MDA reduces this to 10 ng–100 ng, albeit at the cost of completeness in site detection.

Mechanical shearing (sonication) is not the sole approach that allows escaping the RED-dependent bias in PCR-based techniques. One example is the tagmentation-assisted PCR (tag-PCR). Tag-PCR is a method for preparing amplification-based libraries for DNA sequencing based on simultaneous fragmentation and adapter attachment using Tn5 transposase. Originally introduced by Adey et al. (2010), it was designed to simplify and accelerate the construction of shotgun NGS libraries by reducing the protocol steps and processing time. The method utilizes a dimeric Tn5 transposase pre-loaded with a pair of double-stranded adapters containing short 19-bp mosaic ends (ME) derived from IS50 sequences, which are essential for transposition. The loaded enzyme (“transposome”) introduces double-stranded breaks across genomic DNA with near-random distribution while simultaneously attaching sequencing adapters. The initial tagmentation step is universal, while the downstream steps depend on the specific application and sequencing platform. In its original form, limited-cycle PCR is performed using primers that incorporate flow cell adapters and sample barcodes, allowing the preparation of full NGS libraries from as little as 10 pg input DNA in under 30 min.

Tag-PCR provides high coverage and efficiency, though some sequence bias may occur due to Tn5 preferences. Because of its speed and low input requirement, Tag-PCR has been widely used in genomics, including for ISA, transcriptomics, and profiling of rare samples (Hesselberg Løvestad et al., 2023; Kim et al., 2023; Hamada et al., 2018). In the ISA context Tag-PCR protocols typically use one primer targeting the adapter (e.g., Nextera R1) and another primer that is specific to the transgene (or proviral sequence). Several modifications of this method have been developed to reduce nonspecific background amplification.

One such modification is saTag-PCR, which uses a customized Tn5 transposase loaded with two identical adapters (usually Nextera R1). This prevents the amplification of fragments flanked by R1 and R2 (i.e., without the transgene), and the missing adapter is introduced during a second PCR round. Another approach, esTag-PCR, developed by Ryu et al. (2022), utilizes standard commercial Tn5 with dual adapters but modifies the amplification strategy: in the first PCR, only one adapter-specific primer and one transgene-specific primer are used, and no full-length adapter is restored. In the second, nested PCR round, a new internal transgene-specific primer is used, which also carries a TruSeq-compatible adapter. As both PCR rounds depend on gene-specific priming, background amplification is minimized. The method supports multiplexed primer pools targeting both 5′ and 3′ ends of the transgene, allowing the bidirectional mapping of integration sites. esTag-PCR yielded 8–13 times more on-target reads than Tag-PCR and saTag-PCR and successfully validated all tested integration sites by Sanger sequencing. It enables the detection of integration events from as little as 0.2 ng to 0.5 ng of genomic DNA, corresponding to ∼30–150 cells, demonstrating high sensitivity and accuracy.

Another variant of the classic tagmentation-assisted PCR is DIStinct-seq, developed by Kim et al. (2023), which was designed for rapid and efficient genome-wide ISA of lentiviral vectors. DIStinct-seq uses a bead-linked Tn5 transposome (Illumina DNA Prep) that allows up to 500 ng input DNA and reduces sample loss and handling time. The method achieves high sensitivity and reproducibility, detecting 5,000–6,000 unique integration sites per sample with minimal background. DIStinct-seq was applied to study lentiviral integration in CAR-T cells from multiple donors, accurately recapitulating known lentiviral integration biases (e.g., enrichment near TSS, oncogenes, and CpG islands). The method supports the quantitative assessment of clonal composition and longitudinal dynamics of CAR-T cells both *ex vivo* and *in vivo*, confirming associations between integration site location and clonal expansion. A custom bioinformatics pipeline filters out artifacts such as chimeric reads, PCR recombination products, and improper orientations, enabling reliable high-throughput ISA.

Alongside DNA sequence analysis-based methods for proviral integration site mapping, RT-PCR-based techniques emerged to specifically capture transcriptionally active insertions. Valk et al. (1997) developed a targeted approach exploiting the typical outcome of retroviral mutagenesis: when proviruses integrate near proto-oncogenes, their 5′ LTRs often drive aberrant chimeric transcripts fused to host gene exons. By designing an oligo (dT)-adapter primer for cDNA synthesis (anchored to the poly (A) tails of these transcripts) paired with an LTR-specific PCR primer, they selectively amplified only integration-derived chimeric cDNAs and excluded transcriptionally silent insertions. This bypassed the need for genomic DNA processing steps such as restriction digestion and adapter ligation that are inherent to LM-PCR/iPCR. The resulting amplicons, representing LTR–host gene fusions, were directly cloned and sequenced, enabling the rapid identification of oncogenic drivers such as EVI1 in murine leukemias (Valk et al., 1997).

### 3.2 Modern methods with no reliance on PCR

Amplification artifacts and variable efficiency of PCR-based methods can distort quantitative assessment of clonal characteristics of infected cells, complicating the accurate determination of clone prevalence and size. Since these characteristics are often as important as precise IS mapping (sometimes even more so), many recent approaches avoid PCR altogether, aiming to overcome its limitations and provide more reliable quantitative analysis of integration profiles. Several such RED- and PCR-independent pipelines have now been developed.

#### 3.2.1 AFIS-seq

Since 2013, the CRISPR/Cas system has gained broad popularity and widespread application in laboratory practice due to its relatively easy and inexpensive targeting of specific loci. The CRISPR/Cas system has also found application in the ISA field. In particular, van Haasteren et al. (2021) proposed a method called amplification-free integration-site sequencing (AFIS-Seq), which is based on the excision of internal proviral regions using Cas9 RNPs delivered into cells along with guide RNAs targeting sites located within the viral (or vector) genome, approximately 500 base pairs from the LTRs. Host genomic DNA is first isolated using high molecular weight (HMW) DNA extraction to ensure acquired fragment size >50 kb and protected from ligation by dephosphorylation. After Cas9-mediated cleavage, new exposed DNA ends are A-tailed and ligated with Nanopore adapters (in the authors’ study, a 9.4.1 flow cell on a MinION Mk1B sequencer was used), and sequencing proceeds with *in silico* filtering of informative reads containing both host genome and provirus sequences.

When applied to genomic DNA from HEK293T cells transduced with rHIV.VSV-G and rSIV.F/HN, the authors obtained approximately 200,000 reads per vector, each approximately 12 kb in length, indicating up to 285- and 1,612-fold enrichment (for HIV and SIV, respectively), normalized to the number of integrated vector copies. In a direct comparison, the efficiency of unique IS mapping was 4% (HIV) and 3% (SIV) for S-EPTS/LM-PCR and 0.4% and 2%, respectively, for AFIS-Seq, reflecting lower enrichment efficiency of the amplification-free method. However, AFIS-Seq demonstrated higher mapping quality: long reads (∼11 kb) enabled confident identification of IS even in difficult-to-map genomic regions (including repetitive elements), where S-EPTS/LM-PCR failed. Moreover, AFIS-Seq provided simultaneous information on the proviral sequence, clonal composition of infected cell populations, and DNA modifications (e.g., CpG methylation). The method is scalable and can be implemented on higher-throughput Oxford Nanopore platforms such as GridION or PromethION.

The main limitation of AFIS-Seq is the requirement for a large amount of input genomic DNA (∼10 μg), which reduces sensitivity when the proviral copy number (VCN) is low, especially in samples with low infection rates or rare transduced cells.

#### 3.2.2 CReVIS-seq

A year later, in 2021, another CRISPR/Cas-related method was proposed by Kim et al. (2021). In their approach, genomic DNA is mechanically fragmented (by sonication) to generate fragments of approximately 300 base pairs. After end-repair and phosphorylation, the fragments are circularized by self-ligation (this requires a low DNA concentration to reduce concatemer formation). The remaining linear fragments are removed by treatment with T5 exonuclease and DNA circles are re-linearized using Cas9 guided to a site within the LTR. As a result, only fragments containing proviral sequences are cleaved, ensuring the selectivity of the method. The linearized DNA fragments undergo A-tailing (addition of an adenine to the 3′ end) and ligation to sequencing adapters. After several cycles of PCR amplification to enrich the libraries, they are sequenced on high-throughput platforms such as Illumina MiSeq or MiniSeq, using paired-end reads that enable the precise identification of the host–virus junction. Since the target fragments have already been selected and linearized independently of PCR, the subsequent PCR amplification for library enrichment does not introduce the same systematic distortions in the representation of integration sites as observed in PCR-based methods. This method allowed the authors to accurately and clonally identify multiple IS of lentiviral vectors throughout the host genome, even in the presence of multiple integrations and heterogeneous cell populations, and detect circular forms of lentiviral DNA.

The method’s potential limitations are the relatively high input DNA requirement (in the microgram range), its dependence on the presence of an intact sgRNA recognition site within the LTR, a technical constraint on fragment length imposed by the chosen sequencing platform, and the need for careful optimization of circularization conditions to avoid concatemer formation.

#### 3.2.3 Targeted sequence capture for lentiviral integration site identification

Ustek et al. (2012) proposed a targeted sequence capture method for analyzing lentiviral vector integration sites in the human genome without PCR amplification in the step of provirus–host junction retrieval. This method combines hybridization capture of fragmented genomic DNA (100 bp–600 bp), containing viral genome regions (using a set of overlapping probes covering the entire provirus), with subsequent high-throughput sequencing (454 GS FLX pyrosequencing). Viral–host junctions were identified by aligning reads first to the vector and then to the human genome (GRCh37/hg19), thus filtering out nonspecific fragments. This avoids PCR and restriction enzyme biases, enabling accurate mapping of integration sites and reconstitution of provirus structure. The authors identified 203 unique integration sites for the HIV-1-based vector, predominantly in introns and away from CpG islands and transcription start sites.

The advantages, in addition to the absence of PCR and restriction digestion, include high sensitivity for detecting novel integration sites, versatility in analyzing various genomic contexts (genes, TSS, CpG islands, and repetitive elements), and cost efficiency. The limitations, common to all amplification-free methods, are high DNA input (∼500 ng) and low capture efficiency (>90% of reads lack viral sequences), which increase sequencing costs.

### 3.3 Single-cell integration site analysis

Currently, single-cell analysis of integration sites (single-cell ISA) is increasingly being considered useful as it may allow the determination of both the number and precise location of individual proviruses within single cells following transduction with retroviral vectors, providing a potential link between individual integration events and the clonal properties of transduced cells, while overcoming the limitations of bulk analysis, where signals from different cells are averaged. This is particularly relevant in clinical applications of retroviral vectors. In the context of CAR-T cells, a single-cell approach enables the direct correlation of integration sites with the functional state of individual clones as cells differ in survival and proliferative capacity. Similarly, when using integrating vectors in gene therapy, for example in the modification of hematopoietic stem cells or during iPSC reprogramming, single-cell ISA allows linking specific integration events to safe and functionally successful clones and assessing therapeutic gene expression stability and oncogenic risk.

Some of the methods discussed above, such as MIP-seq, have performed reasonably well in this regard, demonstrating sufficient sensitivity to work with individual cells. In this case, the increased sensitivity was achieved through whole-genome nonspecific amplification of DNA from highly diluted samples using MDA.

The EpiVIA method (a modified single-cell ATAC-seq, another variant of tagmentation-based approaches) was proposed by Wang et al. (2020) to simultaneously determine the functional identity of individual CAR-T cells and precisely map lentiviral integration sites, addressing whether integration affects proliferative potential and clonal properties. Tn5 transposase fragments and tags nucleosome-free, accessible regions of chromatin and integrated proviral DNA, generating host–virus chimeric fragments that are sequenced and mapped to a combined reference genome. In an experiment with ∼1,000 cells, 188 integrations were identified in 172 CAR-T cells; in the most favorable conditions—cells containing more than 72,499 unique fragments—integration site detection reached 35%, while proviral reads were detected in 96% of cells, confirming CAR-T identity. In Yan et al. (2023), EpiVIA was applied for additional validation in a limited number of cells from a single patient. The method detected two integration sites within one cell, demonstrating its capability to identify multiple integration events in individual cells (Wang et al., 2020; Yan et al., 2023).

The method has limitations related to the low detection rate of integrations, which is inherent to all droplet-based single-cell protocols, and a strong dependence of sensitivity on sequencing depth and the number of fragments obtained per cell. In addition, PCR amplification of libraries could also potentially introduce bias.

## 4 Conclusion

Accurate methods for identifying integration sites (ISA) remain crucial for assessing the safety of integrating viral vector application and understanding patterns of integration, i.e., studying clonal dynamics of transduced cells. Random (with a certain previously described bias) integration of retroviruses, including lentiviruses, and retroviral vectors into the host cell genome poses a risk of insertional mutagenesis and oncogene activation. Modern ISA methods enable systematic detection of such events, refinement of integration patterns, and assessment of the clonal dynamics of modified cells *in vivo*, thus providing critically important data for preclinical and clinical studies.

The history of ISA methods reflects a transition from limited and labor-intensive approaches to high-throughput and more precise strategies. Early methods, involving the combined use of SB, RED, and molecular cloning, detected integrations primarily at preselected loci and exhibited significant locus-dependent variability in sensitivity. Over time, these were supplemented by local sequencing. The emergence of PCR-based methods, such as inverse PCR (iPCR), LM-PCR, and LAM-PCR, allowed for more comprehensive and unbiased mapping of integration sites across the host genome, although biases related to restriction digestion and amplification remained. LM-PCR and LAM-PCR continue to be used; however, more modern variants, including nrLAM-PCR and Tag-PCR, minimize systematic biases associated with restriction digestion and provide a more uniform representation of integration events. For even more accurate analysis, methods free from major sources of distortion—RED and PCR amplification—have been developed, such as AFIS-seq and CReVIS-seq. Although most contemporary methods, including PCR-dependent techniques, are adapted for high-throughput sequencing, the combination of NGS with relatively “bias-free” approaches provides the most accurate and undistorted representation of various IS.

In the commercial and research sectors, LM-PCR, LAM-PCR, and their modifications (nrLAM-PCR) continue to be applied, being offered by companies such as TaKaRa Bio, Azenta GENEWIZ, and ProtaGene, along with some PCR-free approaches utilizing mechanical DNA fragmentation. A summary comparison of ISA methodologies, aiming to assist in method selection, is provided in Table 1.

**TABLE 1:** Comparison of some modern ISA approaches.

Methodological group	ISA method	Key steps	Distinguishing feature	Advantage	Limitation
PCR-based with RED DNA-shearing	iPCR (Silver and Keerikatte, 1989)	RED DNA shearing → circularization → PCR with outward facing primers annealing to the known provirus sequence → subcloning + Sanger sequencing or adapter ligation + NGS	Amplification of unknown sequences flanking IS, allowing to map IS on the host genome	Being less time- and labor-intensive and more sensitive allows more comprehensive IS identification (genome wide) compared to the earlier methods for preselected loci analysis	Requires empirical condition optimization to prevent concatemerization. RED-dependent bias possibility
	LM-PCR (Pfeifer et al., 1989; Garrity and Wold, 2025; Mueller and Wold, 2025)	RED shearing of genomic DNA → adapter ligation → PCR with both virus-specific and an adapter-specific primers → subcloning + Sanger sequencing or library preparation + NGS	Uses adapters as a known target for primers allowing nonspecific amplification of unknown flanking regions	Genome-wide IS recovery with higher sensitivity and scalability, possibility for clonal assessment	Depends on the distribution of restriction sites in the genome, which can lead to incomplete or biased coverage
	LAM-PCR (Woods et al., 2003; Schmidt et al., 2007; Schmidt et al., 2009)	LA with a biotinylated primer → enrichment using streptavidin-coated magnetic beads (SCB) → synthesis of dsDNA fragments → RED → dsDNA adapter ligation → PCR (nested) with both virus-specific and an adapter-specific primer → Subcloning + Sanger sequencing or library preparation + NGS	LA stage with biotinylated primer enables enrichment by junction containing fragments using streptavidin coated beads	Higher sensitivity than LM-PCR, allowing the detection of proviral sequences at a very low copy number (down to one copy per 100–1,000 cells)	Methodologically complex and still dependent on RE site distributions (RED-dependent bias prone)
PCR-based RED-independent	nrLAM-PCR (Gabriel et al., 2009; Paruzynski et al., 2010)	Linear amplification with a biotinylated primer → enrichment on SCB → ssDNA adapter ligation→ PCR (nested) with both virus-specific and an adapter-specific primer → library preparation + NGS	LA stage with biotinylated primer enables enrichment by junction containing fragments using streptavidin-coated beads. Amplification of fragments with various lengths for the same IS	More uniform detection of integration sites due to the absence of RED stages	Lower sensitivity than LAM-PCR with a small amount of input DNA (still comparable to the LM-PCR)
	s-EPTS/LM-PCR (van Haasteren et al., 2021)	Host DNA mechanical shearing → magnetic extension primer tag selection (EPTS) to remove non-target DNA → adapter ligation → PCR with both virus-specific and an adapter-specific primer → library preparation → NGS	Uses mechanical DNA shearing (instead of restriction digest) and magnetic EPTS selection to remove non-target DNA. An LM-PCR adaptation for NGS sequencing	Eliminates restriction enzyme-associated biases. Offers superior assay performance in terms of quantification, precision, accuracy, and reproducibility Enables fast-track analysis for clinical scenarios	Multi-step PCR protocol requires strict control to minimize quantitative bias and is sensitive to the quality of the input DNA.
	MGS-PCR (Beard et al., 2014; Xu et al., 2013)	Mechanical DNA shearing → End-repair and A-tailing → Ligation of a modified splinkerette adapter with a 3′-T overhang → PCR with LTR-specific primers → library preparation → NGS	Replacement of the standard ligatable adapter with a modified splinkerette bearing a 3′-T overhang; use of nanosized magnetic beads. Utilizes merged paired-end reads for improved localization	Eliminates restriction enzyme biases. Enhances specificity and reduces nonspecific amplification. Improves the accuracy of clonal quantification	Susceptible to quantitative bias (PCR bias) and often requires a relatively high amount of input DNA
	FLEA-PCR (Pule et al., 2008)	LA with a biotinylated primer → enrichment on SCB → attachment of “anchor primer” with a random 3′ tail to the LA products and dsDNA synthesis → PCR → library preparation + NGS	Uses an anchored primer with a randomized 3′ tail to prime dsDNA synthesis	Addresses the bias introduced by RED in standard ISA methods	Can generate multiple amplicons from the same integration site, complicating data interpretation
	MIP-seq (Einkauf et al., 2025)	Whole-genome multiple displacement amplification (MDA) → LM-PCR or nrLAM-PCR/PCR to amplify provirus sequence → library preparation + NGS	MDA usage, paralleled provirus amplification and IS identification for same portions of DNA (from as few as one cell)	Enables the simultaneous identification of integrated proviral sequences and precise IS localization from a single cell	Determined by the ISA method used
	PCIP-seq (Artesi et al., 2021)	Mechanical DNA shearing → circularization → selective linearization with Cas9 → inverse PCR → Nanopore sequencing	Same as in iPCR, + mechanical DNA shearing instead of RED	High-sensitivity ISA due to long reads, precise mapping in complex repetitive regions, etc.	Requires large input DNA (∼3 µg) and empirical condition optimization to prevent concatemerization
	RAISING (Wada et al., 2022)	LA with provirus specific primer → polyAG-tailing → hybridization of a universal oligo-dT adapter to the tail → PCR using universal adapter primer + virus-specific primer → Sanger sequencing/library preparation + NGS	polyAG tail used as a priming site for oligo-DT adapter	Significantly reduces assay time to approximately 3.5 h and lowers costs. It is also highly sensitive	Sensitive to the quality of input DNA and requires a column-based ssDNA purification step, which may affect overall efficiency
	Tagmentation-PCR methods (Hesselberg Løvestad et al., 2023; Kim et al., 2023; Hamada et al., 2018; Ryu et al., 2022)	Tagmentation (Tn5-transposase-mediated simultaneous fragmentation and adapter attachments) → nested PCR with provirus- and adapter-specific primers → library preparation + NGS	Combines DNA fragmentation and adapter ligation in a single step using a transposase	High sensitivity with little input DNA required (as low as 10 pg). Is not time-consumable (it could be done in less than 30 min)	May have some sequence bias due to the preferences of the transposase
	EpiVIA (Wang et al., 2020; Yan et al., 2023)	(Modified scATAC-seq): Tn5 transposase fragments and tags accessible chromatin regions with proviruses and generates host–virus chimeric fragments → library preparation (using PCR) → sequencing and mapping to a combined reference genome	Single-cell joint profiling of chromatin accessibility and lentiviral integration sites. Uses Tn5 transposase instead of RED	Allows simultaneous determination of a cell’s functional state and the precise integration site at the single-cell level. Capable of identifying multiple integration events in individual cells	Limitations: Relatively low detection rate (∼30–40% under optimal conditions), inherent to droplet-based single-cell assays. Sensitivity strongly depends on sequencing depth and the number of fragments captured per cell. PCR amplification during library preparation may introduce quantitative bias
PCR/RED− independent	AFIS-seq (van Haasteren et al., 2021)	HMW DNA extraction (fragment size >50 kb) → dephosphorylation → Cas9 RNP-mediated excision of internal proviral regions → A-tailing of newly exposed ends → adapter ligation + Nanopore sequencing	Uses CRISPR/Cas9 to specifically expose integration junctions from inside provirus, HVW DNA extraction used to obtain long fragments comparable to Nanopore sequencing	Avoids artifacts and quantitative bias from PCR amplification	Requires a large amount of input DNA (∼10 µg), which reduces sensitivity
	CReVIS-seq (Kim et al., 2021)	Mechanical fragmentation (sonication) of DNA → circularization → re-linearization with Cas9 targeted to the LTR →A-tailing, adapter ligation and NGS	Uses CRISPR/Cas9 to precisely re-linearize circularized DNA containing LTRs	Accurately and clonally identifies multiple integration sites	Sensitive to circularization conditions and requires a relatively large amount of input DNA.
	Targeted sequence capture (Ustek et al., 2012)	Mechanical fragmentation (sonication) of DNA → hybridization capture of fragmented genomic DNA with a set of overlapping probes, specific to provirus sequence → library preparation + NGS	Uses hybridization probes to capture provirus-host junctions without PCR amplification	Avoids PCR and restriction enzyme biases; high sensitivity for detecting novel integration sites; versatile in analyzing various genomic contexts	Requires high DNA input (∼500 ng) and has low capture efficiency, increasing sequencing costs
